# 气管腺样囊性癌继发气管鳞癌1例

**DOI:** 10.3779/j.issn.1009-3419.2013.01.11

**Published:** 2013-01-20

**Authors:** 丹 彭, 洪武 王, 存良 蔡

**Affiliations:** 100028 北京，煤炭总医院肿瘤内科 Department of Medical Oncology, China Meitan General Hospital, Beijing 100028, China

## 1 引言

气管腺样囊性癌（tracheal adenoid cystic carcinoma, TACC）在气管癌中发病率较低，近5年我科收治的TACC患者有45例，其中中青年患者多见，我科收治的一例TACC患者3年前经气管镜下消融及粒子植入等治疗后病情好转，今年病情加重，进一步检查发现病理为气管鳞癌，这是我科唯一1例TACC后继发气管鳞癌的病例，现做一报告。

## 2 病历资料

患者，女，33岁，2009年9月出现活动后气短，逐渐加重，偶伴咳嗽，无痰，无发热，未行诊治。2010年6月患者休息时亦感气短症状，右侧卧位明显，2010年7月就诊我院，胸部增强CT提示左主支气管完全阻塞，左全肺不张，阻塞近端隆突上方气管后壁隆起小结节影。纵隔及双侧腋窝可见增大淋巴结（图 1A）。气管镜下见气管内隆突样不规则肿物，累及右主支气管开口，左主支气管被肿物完全堵塞（图 1B），给予镜下二氧化碳冻取及氩气刀烧灼肿物等消融治疗，并取组织活检，病理报告：“气管”腺样囊性癌（图 1C）。2010年8月2日气管镜下于左主支气管内顺利植入碘（125）粒子20枚，中人氟安化疗粒子200 mg，2010年8月10日在气管中下段多点分次植入碘（125）粒子30枚，中人氟安200 mg，2010年8月17日在全麻下经硬镜行Y型气管支架置入术，治疗后患者左肺基本复张，双侧主支气管通畅，气喘症状缓解。此后反复复查气管镜，多次于镜下取组织病理检查均为气管腺样囊性癌，定期复查胸部CT可见左主支气管狭窄较前好转，左肺完全复张（图 1D），病情较平稳。2011年1月患者复查气管镜，镜下见气管上段粘膜充血、水肿，气管腔内较多分泌物（图 1E），在气管镜直视下取出支架。取出支架后见气管下段及左右主支气管内肉芽增生，管腔轻度狭窄。活检病理：炎性坏死中见细菌和不典型增生上皮细胞团，右主支气管粘膜急慢性炎伴急性炎性渗出，局部上皮鳞化和增生，局灶轻度不典型增生。胸部CT提示气管及左右主支气管管壁增厚，增强后肿块呈轻度强化，本次病理未发现气管腺样囊性癌，但胸部CT提示气管肿瘤有进展，故在气管镜下于气管壁增厚处植入碘（125）粒子20枚。2012年5月患者气短再次加重，镜下见气管3区粘膜不规则增厚，左主支气管远端狭窄约60%，于气管下段多次活检，病理回报：鳞状上皮重度不典型增生，鳞状细胞癌（图 1F）。考虑患者气管内病变范围在主气管下段及左主支气管，范围较大，且为管壁型浸润生长，未发现其它远处转移，给予气管镜下光动力治疗，减轻气管狭窄，患者气短症状有所好转。本患者气管鳞癌，因病变范围大，无法手术切除，故在局部治疗缓解症状之后，给予联合全身化疗2个周期，方案（吉西他滨+顺铂+重组人血管内皮抑制素），复查胸部CT肿瘤未见明显进展，疗效评价为稳定，患者气短、咳嗽、咳痰等症状较前有所好转。

**1:**
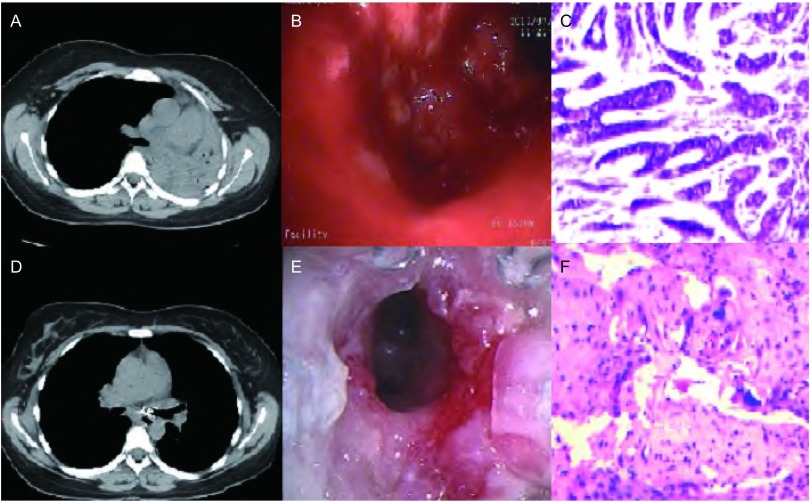
治疗前左肺不张（A）、左主支气管开口完全闭塞（B）、气管腺样囊性癌（HE，×40）（C）; 治疗后左肺复张（D）、左主支气管通畅（E）、气管鳞癌（HE，×40）（F）。 Before treatment, left lung atelectasis (A), left main bronchus obstructed completely (B), tracheal adenoid cystic carcinoma (HE, ×40)(C); After treatment, left lung recruitment (D), left main bronchus Unobstructed (E), tracheal squamous cell (HE, ×40)(F).

## 3 讨论

原发主气管的肿瘤非常少见，占所有肿瘤的0.1%，其中成人中多为恶性，儿童中则良性多发^[1, 2]^。恶性肿瘤最常见的为气管鳞状细胞癌和TACC，两者所占比例约为2/3^[3, 4]^。TACC来源于气管、支气管壁内腺体，其组织结构及生物学行为和涎腺腺体发生的肿瘤类似，多发生于较大气管，约50%发生于主气管，其它主要位于双侧主支气管，其大多数生长呈局限性，并可沿气管支气管壁粘膜下层或神经鞘膜浸润生长，可侵犯临近组织或器官，肿瘤可向管腔内外生长。其病理特点为肿瘤浸润性边界，由内层的导管细胞和外层的肌上皮细胞两种成分组成，呈管状、筛状和实性排列。筛状和管状结构的微囊中含有嗜碱性的黏液，肿瘤细胞小而一致，细胞胞质少，细胞核呈圆形或卵圆形，核深染，核分裂少见^[5]^。本病预后较好，淋巴转移率低，血行转移晚，最常见的部位是肺、胸膜和肝脏，而骨、肾、脾少见。局部复发是TACC主要的致死原因。目前TACC治疗首选手术切除，术后5年生存率为65%-79%，10年生存率为53%-57%。TACC对放疗敏感，术后辅助放疗可提高不完全切除患者的生存率，徐等^[6]^回顾12例TACC患者，单纯手术组4例都出现了局部复发，而术后放疗者6例中无一复发，提示术后放疗可以降低局部复发率。本病对化疗不敏感。气管鳞癌是来源于支气管上皮的一种恶性上皮性肿瘤，可表现角化和/或细胞间桥特征。恶性程度较TACC高。

本患者2009年12月确诊为气管下段及左主支TACC，先后植入碘（125）粒子50枚，中人氟安化疗粒子400 mg，气管及左主支气管狭窄明显缓解。2012年5月气管下段再次不规则增厚，多次病理提示鳞状上皮重度不典型增生，鳞状细胞癌。Wang等^[7]^报道1例气管腺样囊性癌患者手术切除后16年在同一部位发现低分化癌，经伽马刀、放疗等治疗后，在术后27年肿瘤再次复发，病理诊断为气管鳞癌，作者认为在同一部位发生肿瘤病理类型的改变，两者存在一定的联系，因后者恶性程度高，生存期明显降低，故对ACC术后的患者需延长随访时间。本病例中患者TACC治疗3年后，病变处活检未再发现TACC，取而代之的是鳞状细胞重度不典型增生，进而发展成为鳞状细胞癌，时间短，说明两类肿瘤发生发展关系密切。此外我们考虑患者气管鳞癌发生在TACC之后，不能除外放射性粒子及化疗粒子植入后局部作用可能引起的局部组织癌变，尚需进一步观察放化疗粒子对周边组织作用。目前此类病例较罕见，仍需收集更多的病例来寻找两者之间的关系以及从病理生理、分子生物学方面做更深入的研究。

如今肿瘤的治疗已经进入个体化治疗的阶段，本例患者给我们的提示是肿瘤的生长是动态变化的，其诊断与治疗不再是一成不变，它是一个连续的过程，我们的任务是动态监测肿瘤的发展过程，不断地修正诊断，并且调整治疗方案，为每一个患者制定个体化的治疗方案。
